# Nanotech Probes: A Revolution in Cancer Diagnosis

**DOI:** 10.3389/fonc.2022.933125

**Published:** 2022-07-07

**Authors:** Qi Zhang, Kai Hou, Hongbo Chen, Ning Zeng, Yiping Wu

**Affiliations:** Department of Plastic and Cosmetic Surgery, Tongji Hospital, Tongji Medical College, Huazhong University of Science and Technology, Wuhan, China

**Keywords:** nanomaterial, nanotechnology, cancer, diagnosis, nanotech probes

## Abstract

Recent advances in nanotechnologies for cancer diagnosis and treatment have received considerable attention worldwide. Nanoparticles are being used to create nanodrugs and probes to diagnose and treat a variety of diseases, including cancer. Nanomedicines have unique advantages, such as increased surface-to-volume ratios, which enable them to interact with, absorb, and deliver small biomolecules to a very specific target, thereby improving the effectiveness of both probes and drugs. Nanoprobe biotechnology also plays an important role in the discovery of novel cancer biomarkers, and nanoprobes have become an important part of early clinical diagnosis of cancer. Various organic and inorganic nanomaterials have been developed as biomolecular carriers for the detection of disease biomarkers. Thus, we designed this review to evaluate the advances in nanoprobe technology in tumor diagnosis.

## Introduction

Cancer is currently one of the leading causes of death worldwide, with the number of cancer patients expected to increase over the next 50 years as demographic changes, such as population aging and growth, strongly influence cancer incidence and trends across different regions. Assuming that the latest incidence trends of major cancer types continue, the combined incidence of all cancers would double by 2070 (1). Cancer is often fatal, with early diagnosis generally acting as the deciding factor in therapeutic response. Thus, novel cancer prevention strategies and diagnostic tools must be developed to effectively reduce the number of future cancer cases and save more cancer patients.

Cancer is characterized by abnormal cell differentiation and proliferation, uncontrolled growth, invasion, and metastasis, and its occurrence is a complex multi-factor and multi-step process (2). Earlier cancer detection improves survival rate; however, about 50% of cancers are already in advanced stages of pathogenesis at their time of diagnosis (3–5). Early detection of cancer or precancerous lesions allows early intervention attempting to slow or prevent cancer progression and mortality. In addition to a better understanding of risk susceptibility for certain cancers, the biology and trajectory of precancerous and early cancer lesions must be assessed in order to identify secondary diseases that may require intervention. These efforts are best accelerated by early detection research translated into sensitive and specific early detection technologies (6). This need is supported by the observations following the COVID-19 pandemic, which has had a significant impact on cancer patients across the globe. Pandemic responses have resulted in delayed diagnosis and disruptions in treatment and follow-up care, increasing overall infection rates and premature deaths (7–9). This has highlighted the need to reduce the delays in cancer diagnosis associated with traditional diagnostic models and to address the inaccuracy and disruptions in diagnosis caused by COVID-19 (10). In this review, we aim to explore the application of tumor nanotechnology in tumor diagnosis.

## Nanomaterials and Cancer Diagnosis

Nanomedicine is an emerging science often applied in cancer therapy, as it is characterized by tumor-specific drug delivery, conferring a significant therapeutic advantage over traditional interventions (11–15). The integration of imaging and nanoprobes for cancer diagnosis and treatment may facilitate better responses and reduced side effects in normal tissues (14, 16, 17). This means that nano-biosensors are likely to be critical to the development of novel cancer therapies and diagnostics, as they can be used to detect cancer biomarkers, map cancer cells, and monitor metastasis in response to different substrates and conditions (18, 19). Thus, the cancer diagnosis and treatment landscape has expanded considerably over the last decade, propelled by advances in novel therapies and improved diagnostics (14, 16, 17, 20). This recent success in nanomedicine research has also paved the way for accurate diagnosis through the interaction of nanoprobes with specific biological systems (21, 22).

## Nanoprobe Technology

### Fluorescent Probes

Optical tumor detection is becoming more and more common in biomedical research, but its limitations, including light penetration depth and signal attenuation in tissues, need to be overcome (23). Therefore, different optical imaging methods using nanoprobe technology have been developed for application in the fields of fluorescence, phosphorescence, and photoacoustic (PA) imaging. These methods are more sensitive than traditional techniques, providing higher resolution images and making it easier to get information pointing to anoxic areas.

Fluorescent probes are most common in optical sensors. Fluorescent probes bound to selected nanocarriers can produce dense hydrophobic aggregates following self-assembly in aqueous solutions. The probes can be activated by stimulus-specific “off-on” activation, improving the signal-to-noise ratio within the region of interest, resulting in extremely high sensitivity and increased resolution. Many of these probes are used to evaluate multiple tissues and subcellular structures (24).

Aggregation-caused quenching (ACQ) refers to the strong coupling reactions between ground state fluorophores that form a stable non-influenza fluorescence complex with a unique absorption spectrum (25), which is a typical activatable design. Yang et al. (26) took advantage of this simplicity to develop a scalable hypoxic-responsive human serum albumin-triggered nanosystem, consisting of human serum albumin, the near-infrared imaging photosensitizer chlore6, an oxaliplatin precursor, and a hypoxic-sensitive linker, azobenzene 4,4 ‘dicarboxylic acid. When exposed to a hypoxic tumor microenvironment, the nanosystem is cleaved by nitrogen reductase, breaking up the ultra-small human serum albumin aggregates and restoring chlore6 fluorescence, facilitating clear hypoxic imaging (27). Although the ACQ probe is simple to use, the quenching state of the dye largely depends on the assembly state of the nanocarrier. Thus, the status of the ACQs in any given carrier may be influenced by a variety of *in vivo* factors, including protein binding and enzyme degradation, which may lead to decreased selectivity and specificity for tumors.

In addition to general activatable designs, imaging strategies targeting tumor biomarkers *via in situ* luminescence can also be used to effectively image tumors *in vivo*. For example, the HIF-1α-induced transmembrane protein CAIX, which is a biomarker for hypoxic environments, is widely expressed on the surface of hypoxic cells (28). Huang et al. (29) evaluated the application of a CAIX-specific IRDye 800CW probe (CAIX-800) for hypoxia detection in a mouse model of orthotopic nasopharyngeal carcinoma (NPC). Their data revealed that a combination of this dye and fluorescence molecular tomography or computed tomography could be used to accurately locate early-stage NPC tumors, with detection as early as 2 weeks. Lymph node metastases from advanced NPC (6 weeks) were then observed using multispectral PA imaging. Taken together, the results of this study show that molecular conjugation combining appropriate targeting groups and near-infrared dyes can facilitate the selective imaging of specific anoxic analytes *in vivo*.

In addition, anaerobe integration in nanocarriers can also facilitate tumor targeting, as these microbes can only survive in anoxic environments; thus, their inclusion would force aggregation to the hypoxic areas of the tumor. In addition, bacterial migration to hypoxic tumors can also be facilitated by external stimulation. F. Chen et al. (30) attached lipid nanoparticles loaded with indocyanine green to bacterial surfaces to target and ablate hypoxic tumors through photothermal therapy. Fluorescent probes based on nanocarriers are used more and more frequently in tumor diagnosis.

### Phosphorescent Probes

Following photoexcitation, an excited electron passes through the excited triplet state between the systems, inducing a spin transition, which returns a singlet to the ground state and emits photons in the form of phosphorescence. Phosphorescence can then be converted to pO_2_ by calibrating the probe using the Sterne-Volmer equation. Several research groups have gone on to develop phosphorescence probes for various applications, including direct pO_2_ measurement (31, 32). Phosphorescent probes have the advantages of high spatial resolution and direct and reversible pO_2_ quantitative analysis. Yoshihara et al. (33) reported that Ru (II) complexes could be coupled with oligodeoxy nucleotides containing pyrene and nitroimidazole ligands, and that the hydrophilicity of the modified metal complex molecules could facilitate the induction of nanoaggregates, which could then be used to support qualitative analysis of tumor burden. In addition, Liu et al. (34) recently combined complementary imaging technology with nanoparticles to achieve high-quality, reliable, and quantitative hypoxia detection in several different cancer models. In this study, the research team encapsulated benzene-substituted Pd (II) porphyrin (PdTPTBP) into dSPE-PEG 2K phospholipid micelles (Pd-) in MX, to produce a phosphorescent nanoprobe, which could then be applied as a time-resolved lifetime imaging system. By combining the wide field of view of luminescent lifetime imaging with O_2_-sensitive nanoprobes, this group were able to quantify O_2_ localization in tumors and thus identify hotspots for likely tumor formation.

### PA Probes

PA tomography may be another potential approach for addressing the limitations of modern imaging associated with maximum tissue penetration depth. This system works by converting excited light energy to heat/sound energy, facilitating a significant increase in penetration depth while maintaining near-microscopic spatial resolution. PA signals are generated by photon absorption, which causes rapid thermoelastic expansion and sound wave propagation. These pressure waves can be detected by transducers, and PA images can then be produced. These images are characterized by improved tissue penetration depth (35). A recent study (36) used endogenous PA imaging to detect tumor hypoxia in a multicancer model, providing anatomical and functional information on hypoxia. Knox et al. (37) developed a hyper-1-based hypoxic response probe for radiographic PA imaging, which utilized the aza-Bodipy platform and a dialkyl aniline substitutive group, facilitating further oxidation of the system to produce the single n-oxide probe RHP-1. Probe evaluations revealed a two-fold increase in PA 820 nm/PA 770 nm emissions when these sensors were placed under hypoxic conditions. Furthermore, *in vivo* PA imaging revealed that the hypoxic regions of the tumor could be mapped for 3D reconstruction when using a RHP-1 probe. Recently, M. Chen et al. (38) applied the bioreducible N-oxide hypoxia-sensitive probe Hyper-650 with enhanced molar absorption to high resolution PA microscopy. This system facilitated the simultaneous and constant monitoring of vascular sO_2_ and tissue oxygenation, following its combination with endogenous contrast agents. These images revealed that the identified tumor hypoxia center signals were consistent with those identified using traditional sO_2_ imaging, supporting their clinical value.

### Tumor Imaging Based on Dendritic Cells

The tumor microenvironment is characterized by gradual changes in both spatial and temporal heterogeneity, which can be used to explore its diagnostic application in tumors. Fluorescent imaging probes responsive to various conditions, including hypoxia, pH, and protease expression, may be used to evaluate and diagnose specific tumor conditions (39–42). In precision medicine, molecular imaging is primarily used to identify cancer-specific targets, design therapeutic methods, and monitor drug administration responses. DCs, for example, are the most adept antigen-presenting cells, transmitting information to the cells in the adaptive immune system (27, 28). Accurate antigen delivery and effective activation of immune pathways are key to the development of DC anticancer vaccines and successful immunotherapy. However, DC viability, function, and their ability to migrate *in vivo* remain unknown. Superparamagnetic iron oxide (SPION) is an excellent MRI contrast agent that can be easily absorbed by DCs and tracked using MRI. Thus, MRI using SPIONs can provide more anatomical information and more detailed visualization than other methods, but at lower sensitivity (43, 44). To overcome this limitation Y.C. Chen et al. (45) pioneered the development of a novel dual-mode nanoprobe, SPIONIR797, for tracking DC migration *in vivo* and detection *via* a non-invasive combined method. Thus, leveraging the advantages of high sensitivity, high spatial resolution, and relatively simple operation, it is becoming increasingly easy to visualize various diseases and use these imaging techniques to draw critical diagnostic conclusions. Many of these systems focus on a variety of disease models, including tumors, inflammation, and atherosclerosis.

### Other Imaging Nanoprobe Technologies

In addition to the technologies described above, other research groups continue to work on a variety of alternatives. For example, gold, silver, and bimetallic and magnetic nanoparticles are widely used in the manufacture of sensing tools due to their unique optical properties and biocompatibility (22, 46–51). The functionalization of these nanoparticles with different components provides an excellent opportunity to assemble selective and sensitive sensing materials for the detection of various cancer-related biomolecules. This was exemplified in the Zhang et al. (52) study, where a multifunctional ASnFAp : Gd/Tb system was synthesized using a new bionic strategy. Subsequent *in vitro* and *in vivo* experiments then confirmed improved tumor imaging and recognition ability, compared with conventional methods. The generated nanoparticles could be used as drug carriers for tumor imaging and treatment with good tumor recognition, treatment capacity, and superior biocompatibility. Thus, this study supports the very important potential clinical application of nanomaterials in diagnosis.

## Path to Clinical Translation

Given the complexity of the tumor microenvironment and the diversity of analytes that contribute to global hypoxia, there is currently no “gold standard” for measuring tumor hypoxia (53). Nevertheless, several novel methods designed to detect and evaluate tumor hypoxia have been developed, with several entering clinical evaluations. These include oxygen electrodes, electron paramagnetic resonance oximetry, positron emission tomography (PET), and more.

One example is the system created by de Georgia (54), which facilitates pO_2_ evaluation in invasive tumors *via* electrochemical probes. This system has been successfully employed in critical care settings focused on retaining nerve function. However, when the oxygen is completely consumed, the electrode undergoes electrochemical reduction, resulting in reduced signal sensitivity and increasing inaccurate readings. This system is also invasive and can lead to edema and hemorrhage in some tissues. Daimiel et al. (55) developed an alternative technique for repeatable quantitative pO_2_ measurement in tumors using electron paramagnetic resonance oximetry. This non-invasive method requires the use of a paramagnetic probe, which responds to the oxygen in any given environment and evaluates pO_2_ levels by measuring relaxation rates.

In addition, recent developments in 2-nitroimidazole-based radiotracers may facilitate an increase in PET application as an alternative non-invasive technique for detecting tumor hypoxia. Relatively short-lived radionuclides, such as 18F, can be easily bound *via* isotope exchange reactions to produce novel probes. Several studies have evaluated 18F LA-Beled FMISO, pentafluorinated etanidazole, hydrophilic fluoridazole, and arabinosfluorroazomycin as PET probes for hypoxia imaging (56).

However, the development of a new probe must always be followed by preclinical evaluation and scaled-up good manufacturing practices to facilitate clinical trials. Obstacles in each specific area must be overcome before probes can be incorporated into routine clinical practice. Despite all the immediate challenges of clinical transformation, nanoprobe imaging offers significant opportunities to provide improved non-invasive diagnostic tools. Combining optical imaging with other biomedical imaging methods may also facilitate multimodal imaging, providing even better tumor detection.

## Conclusion and Perspectives

Nanoprobes have a unique set of physical advantages, including flexible biocompatibility and pharmacokinetics, and most present various unique nanoscale properties, providing more convenient tumor imaging. Nanostructures can also promote the production of organic dye aggregates. Thus, cancer diagnostic specialists have continued to focus on biomedical sensing and imaging. Nanosensors can also provide a protective substrate to prevent unnecessary interactions within the biological environment and improve *in vivo* circulation time and system delivery. With improved understanding of the basic physical phenomena resulting from nanoconstraints, nanosensors can be designed to facilitate both assembly and disassembly in response to specific physicochemical conditions, providing the possibility for the further development of “smart” responsive nanosensors.

In addition, the high degree of heterogeneity between and within tumors can result in complex diagnostic and evaluation issues, including a large discrepancy in imaging results (57). This highlights the importance of further studies on different tumor types. Considering the temporal and spatial variability of tumors, the development of sensors that can accurately track treatment dynamics in real time is a promising concept. Thus, we believe that nanoprobe sensors are likely to play a fundamental role in our understanding of tumors and are almost guaranteed to become increasingly important in the detection and treatment of various cancers due to their physical, pharmacokinetic, and nanoscopic properties.

## Author Contributions

All authors contributed to the design of the study and writing of the manuscript. QZ, KH, and HC undertook the research. YW and NZ wrote the main manuscript text. QZ and YW revised the article critically for important intellectual content and granted final approval of the submitted version. All authors reviewed the manuscript.

## Conflict of Interest

The authors declare that the research was conducted in the absence of any commercial or financial relationships that could be construed as a potential conflict of interest.

## Publisher’s Note

All claims expressed in this article are solely those of the authors and do not necessarily represent those of their affiliated organizations, or those of the publisher, the editors and the reviewers. Any product that may be evaluated in this article, or claim that may be made by its manufacturer, is not guaranteed or endorsed by the publisher.

## Abbreviations

ACQ, Aggregation-caused quenching; NPC, nasopharyngeal carcinoma; PA, Photoacoustic; PET, Positron emission tomography; SPION, Superparamagnetic iron oxide
